# LPS-Enhanced Glucose-Stimulated Insulin Secretion Is Normalized by Resveratrol

**DOI:** 10.1371/journal.pone.0146840

**Published:** 2016-01-11

**Authors:** Mark K. Nøhr, Anete Dudele, Morten M. Poulsen, Lene H. Ebbesen, Yulia Radko, Lars P. Christensen, Niels Jessen, Bjørn Richelsen, Sten Lund, Steen B. Pedersen

**Affiliations:** 1 Department of Clinical Medicine, Aarhus University, Aarhus, Denmark; 2 Department of Endocrinology and Metabolism C, Aarhus University Hospital, Aarhus, Denmark; 3 Zoophysiology, Department of Bioscience, Aarhus University, Aarhus, Denmark; 4 Department of Hematology, Aarhus University Hospital, Aarhus, Denmark; 5 Department of Chemical Engineering, Biotechnology and Environmental Technology, University of Southern Denmark, Odense, Denmark; 6 Research Laboratory for Biochemical Pathology, Aarhus University Hospital, Aarhus, Denmark; College of Tropical Agriculture and Human Resources, University of Hawaii, UNITED STATES

## Abstract

Low-grade inflammation is seen with obesity and is suggested to be a mediator of insulin resistance. The eliciting factor of low-grade inflammation is unknown but increased permeability of gut bacteria-derived lipopolysaccharides (LPS) resulting in endotoxemia could be a candidate. Here we test the effect of LPS and the anti-inflammatory compound resveratrol on glucose homeostasis, insulin levels and inflammation. Mice were subcutaneously implanted with osmotic mini pumps infusing either low-dose LPS or saline for 28 days. Half of the mice were treated with resveratrol delivered through the diet. LPS caused increased inflammation of the liver and adipose tissue (epididymal and subcutaneous) together with enlarged spleens and increased number of leukocytes in the blood. Resveratrol specifically reduced the inflammatory status in epididymal fat (reduced expression of TNFa and Il1b, whereas the increased macrophage infiltration was unaltered) without affecting the other tissues investigated. By LC-MS, we were able to quantitate resveratrol metabolites in epididymal but not subcutaneous adipose tissue. LPS induced insulin resistance as the glucose-stimulated insulin secretion during an oral glucose tolerance test was increased despite similar plasma glucose level resulting in an increase in the insulinogenic index (IGI; delta_0-15_insulin / delta_0-15_glucose) from 13.73 to 22.40 pmol/mmol (P < 0.001). This aberration in insulin and glucose homeostasis was normalized by resveratrol. In conclusion: Low-dose LPS enhanced the glucose-stimulated insulin secretion without affecting the blood glucose suggesting increased insulin resistance. Resveratrol restored LPS-induced alteration of the insulin secretion and demonstrated anti-inflammatory effects specifically in epididymal adipose tissue possibly due to preferential accumulation of resveratrol metabolites pointing towards a possible important involvement of this tissue for the effects on insulin resistance and insulin secretion.

## Introduction

Obesity and type 2 diabetes are interrelated and the current understanding is that as obesity develops, the body becomes increasingly insulin resistant which can progress into type 2 diabetes. The origin of the developing insulin resistance is not fully known but the concomitantly presence of a chronic low-grade inflammation seems to play an important role [1, 2]. For instance, it was shown decades ago that the proinflammatory cytokine tumor necrosis factor alpha (TNFa) induces insulin resistance [3, 4]. Later, other proinflammatory cytokines such as interleukin 1 beta (IL1b) [5] and interleukin 6 (IL6) [6, 7] have been found to induce insulin resistance in experimental settings.

Metabolic endotoxemia, i.e., endotoxins or LPS in the blood derived from gram-negative bacteria due to increased epithelial permeability, has been suggested as a possible mechanism of low-grade inflammation [8]. Thus, it has been shown that chronic infusion of low-dose LPS commences obesity and insulin resistance in a CD14-dependent manner [8, 9], suggesting a causative link between LPS and development of insulin resistance. However, recent reports have not been able to replicate the effects of LPS on obesity and insulin resistance, and instead suggest that the glucose-stimulated insulin secretion (GSIS) is enhanced by LPS via the GLP-1 pathway [10, 11].

Resveratrol is a polyphenolic compound found in especially red wine, which has been heavily investigated the past decade for its potential anti-inflammatory and anti-diabetic effects [12–18]. Resveratrol suppresses the activation the transcriptional factors NFkB and AP-1, responsible for the induction of cytokines and stress-related stimuli [19, 20]. Previously, resveratrol has been investigated for its effects in acute phase (high dose) LPS stimulation as seen in sepsis [21–23]. However, the effect of resveratrol on chronic low-dose LPS as seen in metabolic endotoxemia, has not previously been studied. Furthermore, resveratrol has been reported as an ameliorating factor on the detrimental effects, such as glucose intolerance and insulin resistance, which is induced by high fat feeding [12, 13]. The molecular mechanism behind resveratrol has for long been debated. It has thus been suggested that resveratrol increases the activity of the intracellular deacetylase sirtuin-1 (SIRT1) either directly [24] or indirectly via AMP-activated protein kinase [25] or effects on phosphodiesterase activity [26], but the precise mechanism is yet to be found. SIRT1 activation is involved in multiple pathways such as PGC1a which is a regulator of mitochondrial biogenesis [13], NFkB involved in inflammatory pathways [27] and PPARg deacetylation and browning of white adipose tissue [28].

The overall aim of this study was to investigate the effect LPS and resveratrol on glucose, insulin and inflammatory status. As low-grade inflammation is seen with obesity, we speculated whether resveratrol could have an ameliorating effect on some of the morbidities.

## Material and Methods

### Mice and diets

Twelve-week old male C57BL/6N mice (Taconic, Ejby, Denmark) were used in the experiments. Mice were allowed free access to food and water and were housed on a twelve-hour light cycle. Mice had free access to a control diet (1324, Altromin, Lage, Germany) or a modified diet consisting of control diet mixed with resveratrol (4 g resveratrol/kg diet) (Evolva, Copenhagen, Denmark) and processed into pellets. Protocols were performed in accordance with the European Communities Directive of 24 November 1986 (86/609/ECC) and approved by the Danish Council for Animal Experiments and conducted under license no. 2013-15-2934-00899.

### Experimental design

**Fig 1:**
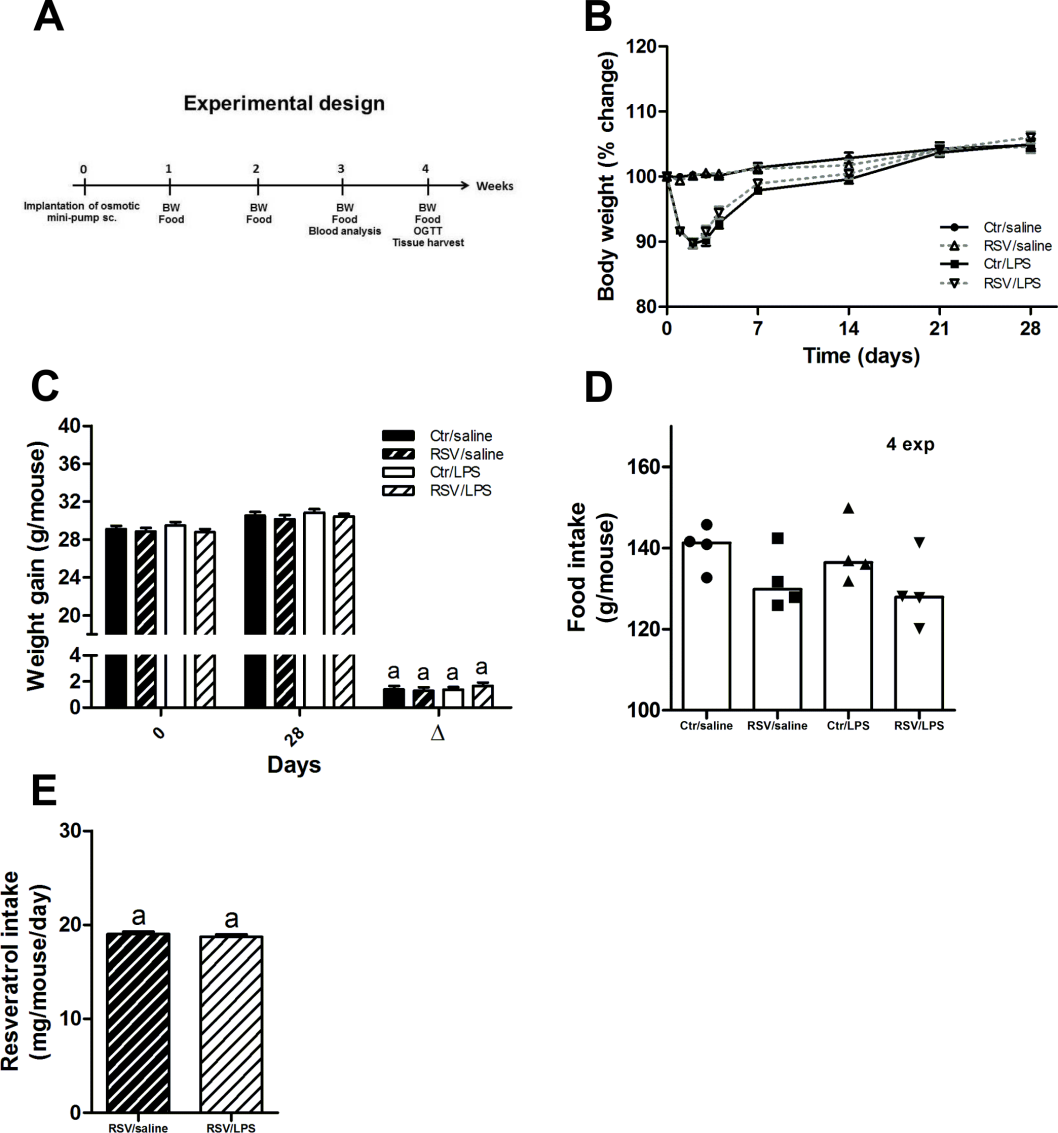
Body weight and food intake. (A) Schematic overview of the research design. (B) Body weight during the course of the experiment in mice treated with control diet and saline (Ctr/saline), resveratrol diet and saline (RSV/saline), control diet and LPS (Ctr/LPS) and resveratrol and LPS (RSV/LPS) (n = 28–29 per group). (C) Total weight gain expressed in g/mouse after 28 days of treatment (n = 28–29 per group). (D) The total food intake during the experimental period of 28 days in four independent experiments. (E) Average daily resveratrol consumption. Data are presented as means ± SEM. Means with different superscript letters are significantly different at P < 0.05 according to post-hoc ANOVA or unpaired t-test.

Mice, anesthetized with a mixture of Hypnorm/midazolam (0.079 mg/ml fentanyl citrate + 2.5 mg/ml fluanisone + 1.25 mg/ml midazolam), were subcutaneously implanted with osmotic mini-pumps (Model 2004, Alzet, Cupertino, CA) infusing either vehicle (saline) or low-dose LPS (*Escherichia* coli 055:B5, L2630, Sigma-Aldrich) for the duration of 28 days (Fig 1A). Initially, we used a dose of LPS at 300 ug/kg/day, which has been previously published [8], but did not observe any effect compared to saline infused mice in relation to insulin secretion. Thus, we doubled the dose to 600 ug LPS/kg/day and saw a similar degree of inflammation of the liver, as was reported by Cani et al. [8]. The mice were divided into four groups: 1) Ctr/saline–control diet with saline-filled pumps, 2) RSV/saline–resveratrol diet with saline-filled pumps, 3) Ctr/LPS–control diet with LPS-filled pumps and 4) RSV/LPS–resveratrol diet with LPS-filled pumps. Body weight was measured daily the first week after surgery and hereafter weekly. Food intake was measured weekly. Following 28 days of treatment, mice underwent oral glucose tolerance test (OGTT) [29]. Mice were euthanized under anesthesia (Hypnorm/midazolam) by cervical dislocation. Tissues were harvested after a 3–5 hour fast and snap frozen in liquid nitrogen for later quantitative polymerase chain reaction qPCR analyses. The following tissues were harvested: liver, epididymal and subcutaneous adipose tissue, muscle (gastrocnemius).

### Oral glucose tolerance test

Following a 5 hour fast in new cages, mice were administered an oral dose of 2 g/kg glucose from a 50% glucose solution. Blood glucose was measured from tail vein blood at 0, 15, 30, 60 and 120 min after glucose administration using a hand-held glucometer (Contour XT, Bayer, Leverkusen, Germany). Furthermore, 75 ul blood samples were drawn at time points 0 and 15 min in heparin-coated capillary tubes, centrifuged and snap frozen in liquid nitrogen for later insulin measurements.

### Gene expression analysis

Total RNA was isolated from liver, muscle and adipose tissue using TRIzol® Reagent (Life Technologies, Carlsbad, CA) according to manufacturer’s protocol. The concentration and purity of the RNA was measured by absorbance at 260 and 280 nm. The integrity of the RNA was evaluated by gel electrophoresis. Reverse transcriptase PCR was performed using Verso^TM^ cDNA Synthesis Kit (Thermo Scientific, Waltham, MA). cDNA was run in duplicates against primer pairs (Table 1) on LightCycler480 (Roche, Basel, Switzerland) using KAPA SYBR^®^ FAST qPCR Kit (Kapa Biosystems, Wilmington, MA). Data are shown as relative copy number compared to housekeeping gene calculated by the Advanced Relative Quantification method in LightCycler480 software v. 1.5 and presented as fold change compared to control. *Polr2a* was used as housekeeping gene on liver and muscle samples whereas *Gadph* was used as housekeeping gene in adipose tissue. All housekeeping genes were tested and had a similar expression level between the four groups. Primer pairs were designed using QuantPrime [30].

**Table 1:** Primers used for qPCR analysis.

Gene	Primer	Sequence (5’ -> 3’)
***Adiponectin***	Forward	CTGGAGACCCGCGTCACTG
	Reverse	TAGGTGAAGAGAACGGCCTTG
***Cd14***	Forward	TGAAGCCTTTCTCGGAGCCTATC
	Reverse	ACGCTCCATGGTCGGTAGATTC
***Gadph***	Forward	TTGATGGCAACAATCTCCAC
	Reverse	CGTCCCGTAGACAAAATGGT
***Glut4***	Forward	AACCAACTGGCCATCGTCATT
	Reverse	GCAGTGGCCACAGGGTAGC
***Hsl***	Forward	AAGGATCGAAGAACCGCAGTCG
	Reverse	TGTGTGAGAACGCTGAGGCTTTG
***Il1b***	Forward	CCTGTGTAATGAAAGACGGCACAC
	Reverse	ATTGCTTGGGATCCACACTCTCC
***Irs1***	Forward	ACTATGCCAGCATCAGCTTCCAG
	Reverse	TCTGCTGTGATGTCCAGTTACGC
***Irs2***	Forward	ATGCAAGCATCGACTTCCTGTCC
	Reverse	GCTGGTAGCGCTTCACTCTTTC
***Pgc1a***	Forward	CCGTAAATCTGCGGGATGATGGAG
	Reverse	TCAAGAGCAGCGAAAGCGTCAC
***Polr2a***	Forward	TCCTGGTGAAGACAATGAAGG
	Reverse	TCATAGACATGCGTAAGCCG

### Western blot analysis

Protein extraction and western blot analysis were performed as previously described [31]. Primary antibodies against GLUT4, AS160, SDHA, glycogen synthase, AKT (isoform 2), cytochrome c, HSP60 and pyruvate dehydrogenase were used. Primary and secondary antibodies, dilution and source can be found in S1 Table. Data were calibrated to an internal control and normalized to total protein.

### Biochemical analyses

Insulin was measured in duplicates using ultra-sensitive mouse ELISA kit (90080, Crystal Chem, Downers Grove, IL) according to manufacturer’s instructions.

Adiponectin was measured using a commercial available ELISA kit according to manufacturer’s protocol (ELM-Adiponectin, RayBiotech, Norcross, GA).

For liver triglycerides measurements, 50 mg liver was weighted and added 125 ul ethanolic KOH in microfuge tubes. Samples were incubated overnight and added 175 ul H_2_O:EtOH (1:1), centrifuged 5 min at 5000 rpm and the supernantant was moved to new tubes. 100 ul EtOH was added, vortexed and 200 ul was moved to new tubes and added 215 ul 1M MgCl_2_. Samples were centrifuged, moved to new tubes and measured for triglycerides.

Free fatty acids were measured by a commercial available kit according to the supplied instructions (NEFA-HA(2), Wako, Neuss, Germany).

### Leukocyte count analysis

Non-fasted blood was collected from the tail vein in pre-chilled EDTA tubes and stored on ice. Samples were analyzed in duplicates for leukocyte count on a hematology analyzer (XP-300, Sysmex, Ballerup, Denmark).

### Resveratrol measurement by liquid chromatography-mass spectrometry

100 mg of frozen samples were homogenized in 1.5 ml microtubes with pestiles (VWR^TM^ Pestle&Microtube, Argos Technologies, United Kingdom) with 200 ul of a solution of 1.5 M formic acid methanol (95:5, v/v), then 1 ml of the same solution was added to the microtube and processed in vortex (Vortex Mixer, Hounisen, Denmark) for 2 min prior centrifugation at 13 400 rpm at room temperature for 30 min. The procedure was repeated one time with 1 ml of a solution of 1.5 M formic acid methanol (95:5, v/v) and two times with 1 ml of a solution of 1.5 M formic acid methanol (20:80, v/v). Pooled supernatants were collected and evaporated to dryness under reduced pressure in ScanVac Speed Vacuum Concentrator (Thermo Scientific). The residue was reconstituted in mobile phase (acetonitrile-water (5:95) v/v) and filtered using syringeless filter device, 0.2 um pore size (Whatman).

Liquid chromatography-mass spectrometry (LC-MS) analysis were performed using LTQ XL (Linear Quadrupole 2D Ion Trap Mass Spectrometer, Thermo Scientific, CA, USA) mass spectrometer operating in electrospray ionization (ESI) negative mode and attached to an Accela HPLC system. Settings for the mass spectrometer were 45, 3, and 0 (arbitrary units) for sheath, auxiliary, and sweep gas flow rates (N_2_), respectively, a spray voltage of 1.10 kV, and a capillary temperature of 350°C. The settings for capillary voltage and tube lens voltage were 3 V and 90 V, respectively. Resveratrol metabolites (*trans*-resveratrol-3-*O*-sulfate, *trans*-resveratrol-sulfate-glucuronide, *trans*-resveratrol-3-*O*-glucuronide, *trans*-resveratrol-4´-*O*-glucuronide, *trans*-resveratrol-3,4´-*O*-disulfate) were separated by a solvent gradient with aqueous formic acid (0.1%, pH 2.5) as solvent A and 100% acetonitrile as solvent B on a Kinetex C18 reverse-phase column (100 mm length, 2.6 mm internal diameter, 1.7 μm particle size; Phenomenex) protected by a precolumn. Solvent gradient: 0 min 5% B, 2 min 5% B, 8 min 30% B, 11 min 95% B, 14 min 95% B and then equilibrating the column at 5% B for 5 min, the flow rate was 0.4 ml/min and the column temperature was 25°C. Glucuronides and sulfates were quantified by an external standard calibration curve of *trans*-resveratrol-3-*O*-β-D-glucuronide and *trans*-resveratrol-3-*O*-sulfate respectively, which were isolated from human urine according the procedure described by Radko *et al*. [32]. The structure of metabolites was identified based on their full scan MS and MS/MS spectra generated in negative ESI. Limit of detection of metabolites was 0.017 and 0.025 ug/g tissue; limit of quantification was 0.018 and 0.032 ug/g tissue for sulfates and glucuronides, respectively.

### Statistical analysis

Data are presented as mean ± SEM. Differences of means were calculated by one-way ANOVA followed by Newman-Keuls post hoc test or unpaired t-test where appropriate. OGTT and insulin levels over time were evaluated by two-way ANOVA followed by Bonferroni post hoc test. Area under the curve (AUC) was calculated using the trapezoidal rule. The insulinogenic index (IGI) was calculated as the initial insulin secretion (delta_0-15_Insulin) divided by the initial glucose rise (delta_0-15_Glucose) following oral administration of glucose. Means were considered significantly different when P < 0.05. Data were analyzed using GraphPad Prism 5.01.

## Results

### Body weight and food intake

In the first few days following implantation of osmotic mini-pumps, LPS mice regardless of resveratrol dropped (≈ 10%) in body weight (Fig 1B). After 28 days of treatment, no differences in body weight were seen between the groups (Fig 1C). Total food intake was evaluated after the entire treatment period (28 days) in four separate experiments. Generally, in the four experiments, resveratrol reduced the food intake independently of LPS (Fig 1D). The food consumption by the two resveratrol groups resulted in a daily oral dose of ≈ 19 mg resveratrol/mouse (Fig 1E).

### LPS induces increased GSIS

**Fig 2:**
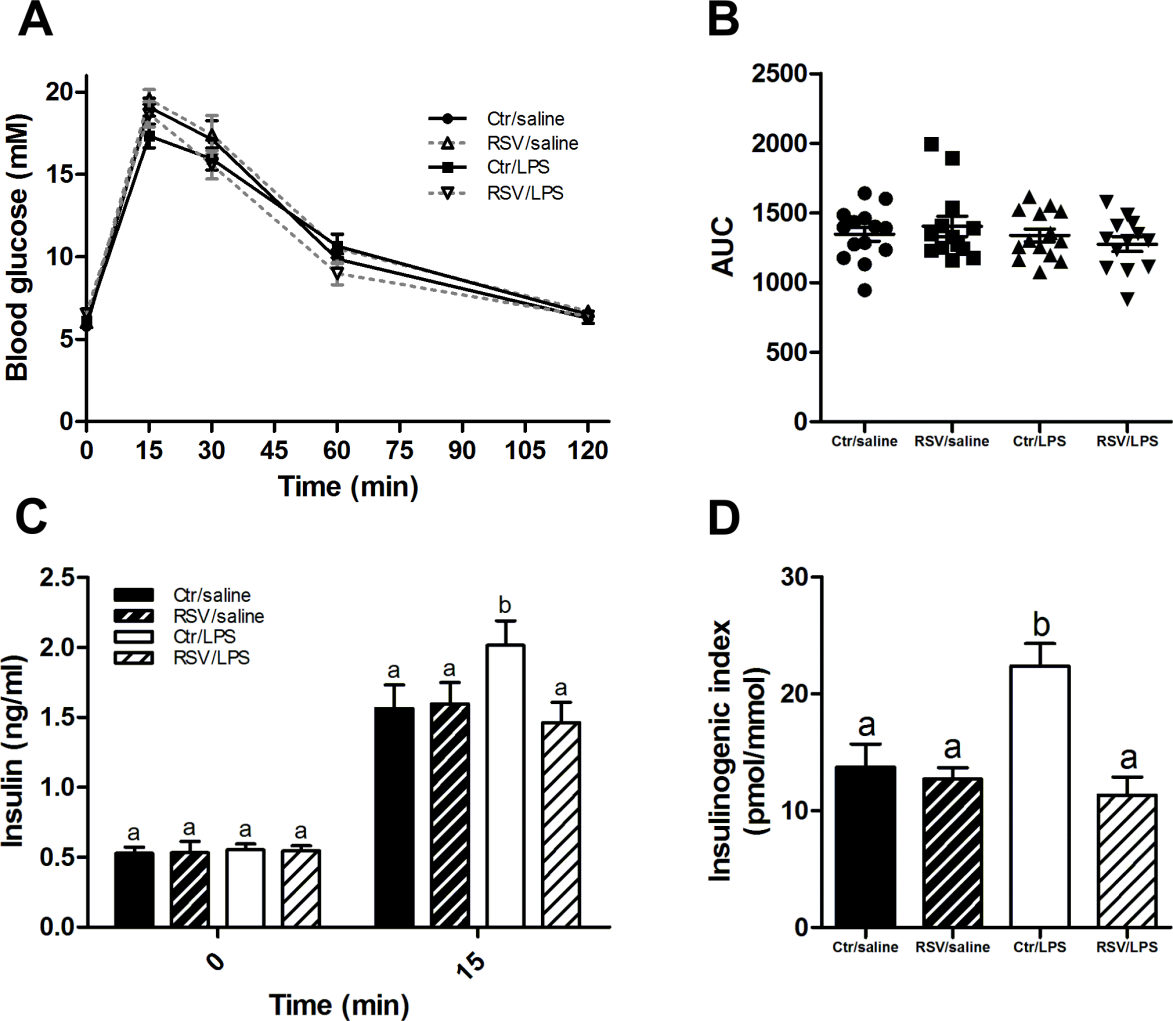
LPS induce enhanced GSIS and is reversed by resveratrol. (A) Oral glucose tolerance test (OGTT) in mice treated with LPS and/or resveratrol (n = 13–14 per group). (B) Area under the curve of (A) for each treatment group. (C) Insulin concentrations 0 and 15 minutes after oral administration of a glucose dose (n = 12–15 per group). (D) The insulinogenic index (delta_0-15_Insulin/delta_0-15_Glucose) was calculated for each treatment group (n = 12–15). Data are presented as means ± SEM. Means with different superscript letters are significantly different at P < 0.05 according to post-hoc ANOVA.

To evaluate the effect of LPS and resveratrol on glucose metabolism, mice underwent an OGTT after 28 days of treatment. In contrast to the original finding [8], LPS-treatment did not cause significant glucose intolerance following an oral glucose bolus compared to control mice (Fig 2A). Area under the curve for the blood glucose did not show any differences between the groups (Fig 2B). However, although fasting insulin levels were similar between groups (Fig 2C), LPS-treated mice had ≈ 29% increased insulin levels 15 min after glucose administration (P < 0.05 vs Ctr/saline). Mice treated with both LPS and resveratrol (RSV/LPS) did not experience the same increase in insulin (P < 0.01 vs Ctr/LPS). The IGI, as a measure of beta-cell function [33, 34], was increased ≈ 63% in LPS-treated mice compared to control animals (P < 0.001 vs Ctr/saline) (Fig 2D) indicating increased GSIS. Resveratrol restored the LPS-induced increased GSIS (P < 0.001 vs Ctr/LPS).

### Effects of LPS and resveratrol on inflammatory status

Next, to evaluate the inflammatory status, whole blood leukocytes were quantified and spleens were weighted. Furthermore, gene expression of the inflammatory markers TNFa, IL1b and the macrophage marker, CD14, were measured by qPCR analysis.

#### Systemic

**Fig 3:**
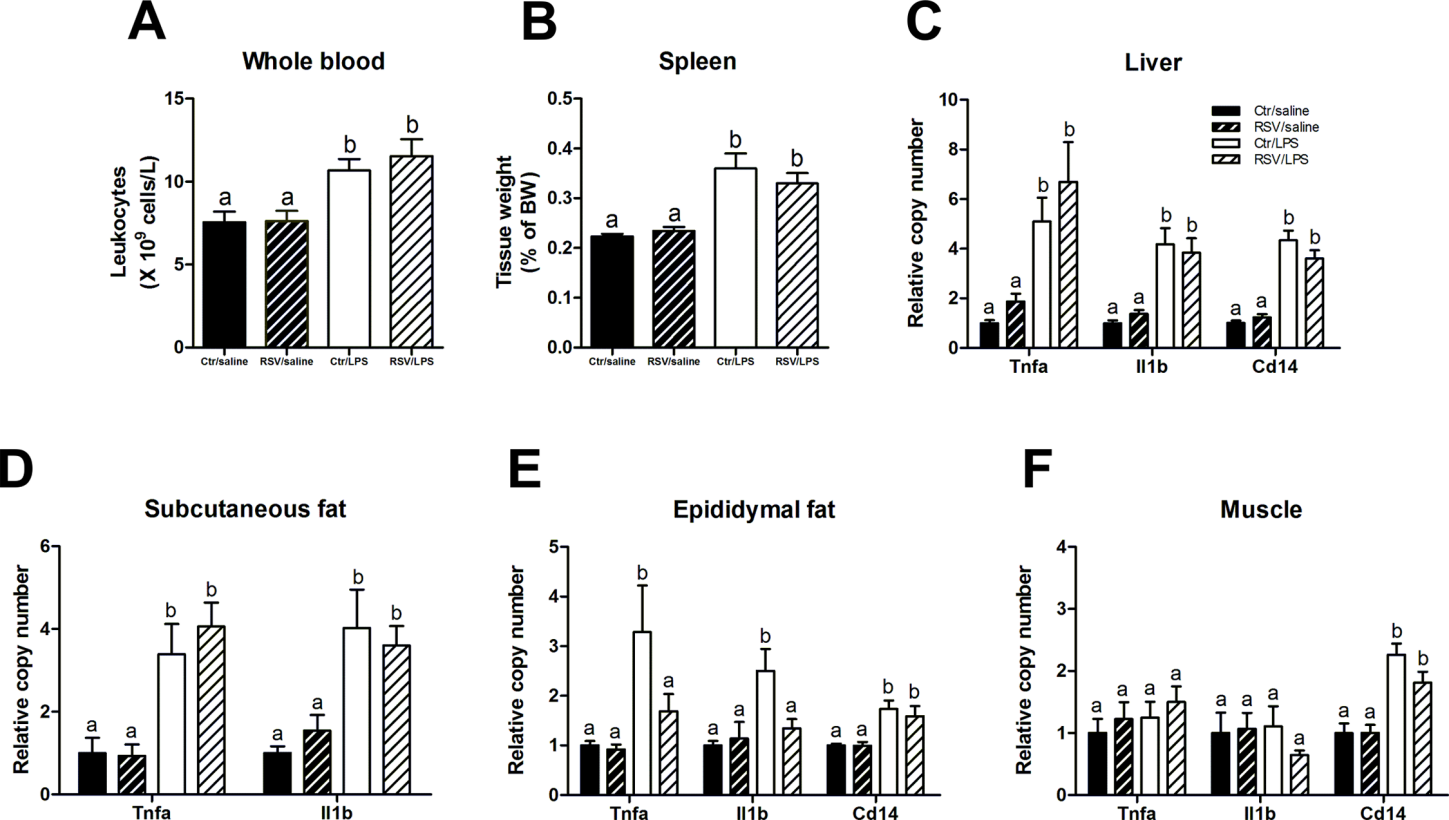
Resveratrol only reduces LPS-induced inflammation in epididymal adipose tissue. (A) Total leukocyte count of whole blood in mice treated with LPS and/or resveratrol (n = 10 per group). (B) Spleen weights as percentage of body weight (n = 10 per group). (C, D, E) qPCR analyses of gene expression of the pro-inflammatory cytokines TNFa, Il1b and the macrophage marker CD14 in liver (C; n = 8–10 per group), subcutaneous (D; n = 10 per group) and epididymal adipose tissue (E; 7–10 per group) and skeletal muscle (F; n = 9–10 per group). Data are presented as means ± SEM. Means with different superscript letters are significantly different at P < 0.05 according to post-hoc ANOVA.

LPS-treated mice had ≈ 41% increased leukocytes in the blood compared to control mice (P < 0.05 vs Ctr/saline) and without any reductive effect of resveratrol (Fig 3A). Also, spleens were enlarged by ≈ 61% in LPS-treated mice compared to controls (P < 0.001 vs Ctr/saline) without any effect of resveratrol (Fig 3B).

#### Liver

LPS increased gene expression of the pro-inflammatory cytokines TNFa (5–6 fold, P < 0.05), IL1b (4 fold, P < 0.001) and CD14 (4 fold, P < 0.001) in the liver (Fig 3C) but there were no anti-inflammatory effect of resveratrol (Fig 3C).

#### Adipose tissue

LPS increased Tnfa and Il1b expression in both subcutaneous and epididymal adipose tissue (Fig 3D and 3E). However, whereas resveratrol had no effect on inflammation in the subcutaneous fat, it exhibited pronounced anti-inflammatory effect in epididymal fat. The decreased inflammation by resveratrol in epididymal fat was not due to decreased macrophage infiltration as the Cd14 expression was unaltered (Fig 3E). We measured the concentration of resveratrol metabolites by LC-MS in epididymal and subcutaneous adipose tissues to see if the there was an alteration of distribution. Interestingly, we found that only epididymal adipose tissue contained measurable amounts of resveratrol metabolites, whereas subcutaneous adipose tissue, except for small amounts of *trans*-resveratrol-sulfate-glucuronide, did not contain resveratrol metabolites (Table 2).

**Table 2:** Resveratrol metabolites in epididymal and subcutaneous adipose tissues.

Adipose tissue	*Trans*-resveratrol-3-*O*-sulfate	*Trans*-resveratrol-sulfate-glucuronide	*Trans*-resveratrol-3-*O*-glucuronide	*Trans*-resveratrol-4´-*O*-glucuronide	*Trans*-resveratrol-3,4´-*O*-disulfate
**Epididymal**	0.035±0.011	0.73±0.14	0.34±0.10	0.043±0.003	0.033±0.009
**Subcutaneous**	nd	0.022±0.003	nd	nd	nd

Data are presented as mean values (μg/g tissue) ± SEM. nd: not detected.

#### Muscle

LPS increased CD14 expression but did not induce inflammation measured by TNFa or Il1b expression. Resveratrol had no effect (Fig 3F).

### Resveratrol ameliorates the LPS-induced down-regulation of adiponectin specifically in subcutaneous adipose tissue

**Fig 4:**
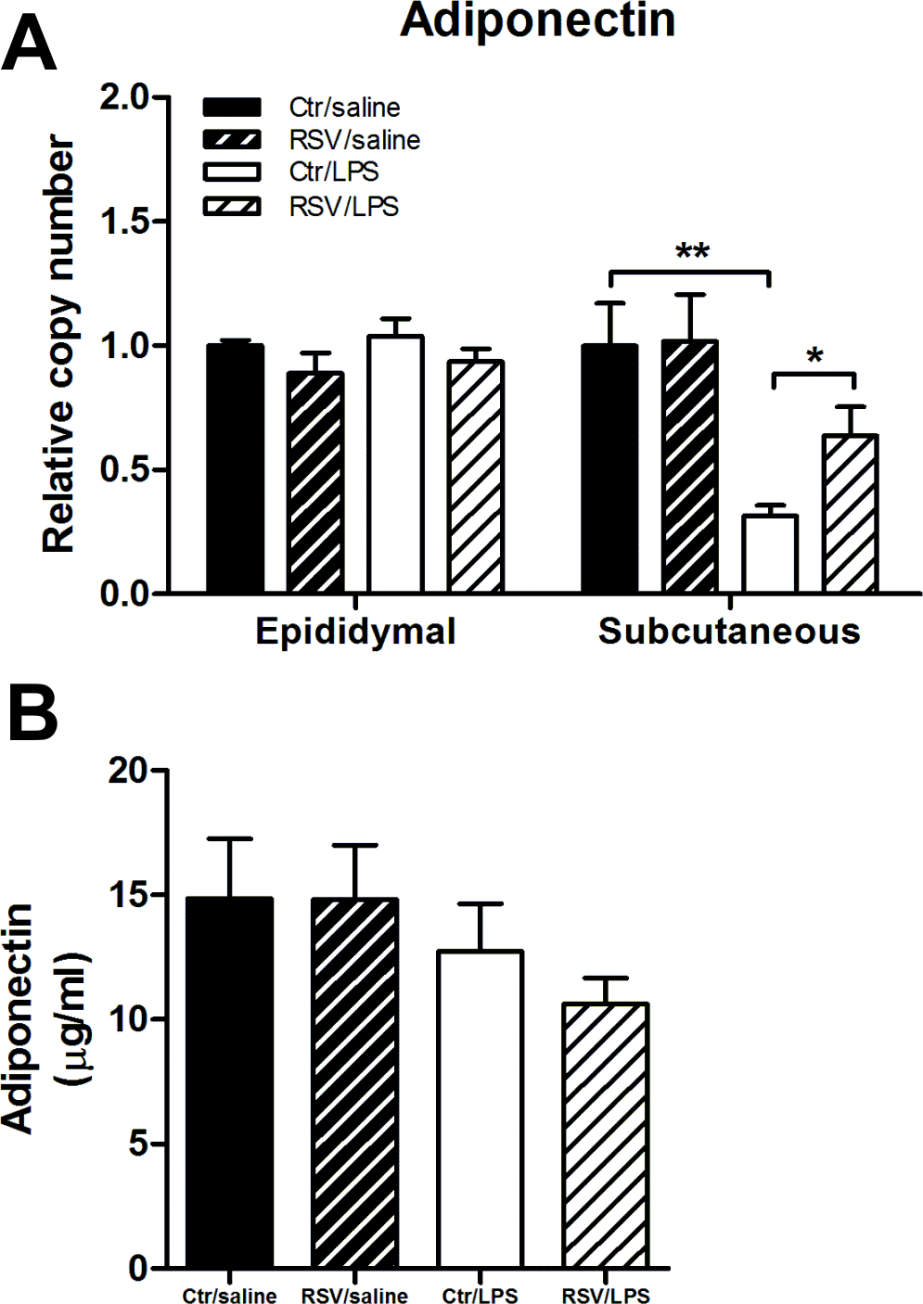
Effect of resveratrol and/or LPS on adiponectin expression. (A) Adiponectin expression was measured by qPCR analysis in epididymal and subcutaneous adipose tissue (n = 9–10 per group). (B) Plasma values of adiponectin (n = 9–10 per group). Data are presented as means ± SEM. *P < 0.05, **P < 0.01 according to unpaired t-test.

As LPS have previously been described as an inducer of insulin resistance [8], we next tested several key pathway molecules known to influence insulin sensitivity. Adiponectin is a peptide hormone secreted from adipose tissue and has a positive effect on the insulin sensitivity [35]. LPS decreased the adiponectin mRNA expression in the subcutaneous adipose tissue which was partly rescued by concomitant resveratrol treatment (Fig 4A). In epididymal adipose tissue, LPS did not influence adiponectin expression (Fig 4A). In plasma, there was a trend towards reduced plasma levels of adiponectin by LPS (albeit not statistically significant) (Fig 4B).

### LPS and resveratrol effects on insulin signaling pathway genes and proteins in epididymal fat and skeletal muscle

Genes such as *Glut4* or *Hsl* in epididymal adipose tissue and *Glut4*, *Irs1*, *Irs2* and *Pgc1a* in skeletal muscle, known to play a role in insulin signaling, were investigated by qPCR analyses. With the exception of a borderline significance of *Hsl* (P = 0.07), none of the genes were affected by resveratrol or LPS (Table 3). Furthermore, as skeletal muscles accounts for up to 80% of the glucose uptake [36], this tissue was investigated by Western blot analysis for protein expression of GLUT4, glycogen synthase, AS160, cytochrome c, pyruvate dehydrogenase, SDHA and HSP60, but no significant changes were seen (S1 Fig). AKT (isoform 2) showed a tendency towards decreased protein expression by resveratrol (S1 Fig), which is a characteristic of negative feedback upon continuous insulin signaling [14].

**Table 3:** Liver and plasma values and gene expression of muscle and epididymal fat.

	Ctr/saline	RSV/saline	Ctr/LPS	RSV/LPS	*P* value
**Liver**					
**Triglycerides (mg/g)**	6.00 ± 0.74^a^	6.10 ± 0.50^a^	4.69 ± 0.44^a^	4.14 ± 0.50^a^	.04
**Plasma**					
**FFA (mM)**	0.84 ± 0.05^a^	0.77 ± 0.03^a^	0.69 ±0.06^a^	0.73 ± 0.04^a^	.13
**Gene expression (au)**					
**Adipose tissue**					
***Glut4***	1.00 ± 0.07^a^	1.07 ± 0.15^a^	1.05 ± 0.11^a^	0.98 ± 0.08^a^	.92
***Hsl***	1.00 ± 0.06^a^	0.93 ± 0.09^a^	1.23 ± 0.16^a^	0.80 ± 0.09^a^	.07
**Muscle**					
***Glut4***	1.00 ± 0.04^a^	1.05 ± 0.04^a^	1.03 ± 0.03^a^	1.03 ± 0.03^a^	.79
***Irs1***	1.00 ± 0.06^a^	1.14 ± 0.06^a^	1.07 ± 0.09^a^	0.94 ± 0.06^a^	.24
***Irs2***	1.00 ± 0.07^a^	1.01 ± 0.12^a^	0.92 ± 0.06^a^	0.89 ± 0.09^a^	.72
***Pgc1a***	1.00 ± 0.15^a^	1.00 ± 0.15^a^	0.83 ± 0.10^a^	0.75 ± 0.13^a^	.47

Different superscript letter denotes significance at P < 0.05 between groups according to post-hoc ANOVA. Abbreviations: Au: arbitrary units; BW: body weight; Ctr: control; FFA: free fatty acid; Glut4: glucose transporter type 4; Hsl: hormone-sensitive lipase; Irs1: insulin receptor substrate 1; Irs2: insulin receptor substrate 2; LPS: lipopolysaccharide; Pgc1a: peroxisome proliferator-activated receptor gamma coactivator 1-alpha; RSV: resveratrol.

Liver triglycerides were slightly reduced by LPS-treatment without any effect of resveratrol (Table 3). Also, plasma free fatty acids were not altered by LPS or resveratrol-treatment (Table 3).

## Discussion

Low-grade inflammation is a key component of obesity and has previously been suggested to be induced by LPS-leakage through the gut epithelium [8]. In the present study, LPS treatment did not cause significant glucose intolerance during an OGTT (Fig 2A). However, the GSIS was elevated by LPS without affecting the blood glucose indicating an induction of insulin resistance. Furthermore, resveratrol restored the elevated LPS-induced GSIS (Fig 4D). LPS-treatment caused inflammation as the inflammatory markers *Tnfa* and *Il1b* were elevated in liver and subcutaneous and epididymal adipose tissues. Also, LPS-treated animals had increased leukocyte numbers in the blood and enlarged spleens, pointing towards an increased inflammatory state (Fig 3). Surprisingly, resveratrol showed a mixed picture of its conceivable anti-inflammatory function. Actually, resveratrol only reduced inflammation in epididymal adipose tissue whereas both liver, blood leukocytes, spleens and subcutaneous fat were unaffected (Fig 3).

In agreement with recent publications [10, 11], LPS enhanced the GSIS. Nguyen and colleagues [10] showed that the enhancement of GSIS by LPS could be traced back to an increased GLP-1 secretion. GLP-1 is an incretin hormone released from the L-cells in the gut, which potentiates the insulin secretion from the beta-cells in the presence of glucose [37]. The physiological relevance for having increased GSIS during inflammation is of a complex nature and poorly understood. First, having a tight glucose control during endotoxemia and disease seems to be important for the body, which is also a predictor of the clinical outcome in critically ill patients [38, 39]. Second, insulin itself could have anti-inflammatory effects and is thus released in order to counteract the effect of LPS. Indeed, constant insulin infusion during normoglycemia decreases inflammation during endotoxemia in animals [40, 41] in a PI3K/Akt-dependent manner [42]. To further complicate the picture, a study by Ceasar *et al*. [43] demonstrated that monocolonisation with either *E*.*coli* or an isogenic strain with reduced LPS immunogenicity in mice resulted many of the same effects, e.g. increased adiposity, glucose intolerance and insulin resistance, even though LPS plasma concentration and inflammation was significantly reduced. Needless to say, much more data are required in order to elucidate the role of LPS in the context of metabolic disease.

It was unexpected to find that resveratrol did not work uniformly as an anti-inflammatory agent, which has been showed previously in various cell cultures [44–46]. Actually, anti-inflammation was specifically seen in epididymal adipose tissue and not liver, skeletal muscle, leukocyte numbers or even subcutaneous adipose tissue (Fig 3). However, this is in agreement with a recent report stating that resveratrol only has an effect in visceral adipose tissue in high-fat fed monkeys, leaving the subcutaneous fat unaffected and inflamed [14]. This is very interesting, as especially visceral adipose tissue inflammation has long been correlated with the development of metabolic syndrome [47, 48]. To investigate whether the mixed anti-inflammatory properties is a result of altered tissue distribution of resveratrol, we quantified resveratrol metabolites in epididymal and subcutaneous adipose tissues by LC-MS. This analysis revealed that resveratrol metabolites are only measurable in visceral adipose tissue with no metabolites (except from small amounts of *Trans*-resveratrol-sulfate-glucuronide) found in subcutaneous adipose tissue (Table 2). So our LC-MS measurement of resveratrol metabolites in the two adipose tissue depots might offer an explanation for the more pronounced effect of resveratrol in visceral adipose tissue. *Cd14* expression, which is a marker of macrophage infiltration, was unaltered (Fig 3E) suggesting that resveratrol does not affect the actually number of residual macrophages, but instead shift their phenotype into a more anti-inflammatory state (M2 macrophage) in the epididymal adipose tissue. Despite the mixed anti-inflammatory properties in various tissues, resveratrol did reverse the LPS-induced increase in GSIS.

Surprisingly, we saw that resveratrol caused a small decline in the food consumption during the treatment period without affecting the overall weight gain/loss (Fig 1C and 1D). One explanation could be due to the relative small reduction in food intake (≈ 7%, Ctr/saline vs RSV/saline) is not sufficient to detect alterations in body weight in the matter of a relative short treatment period (28 days). In a recent study, high-fat feeding (70% fat) for one month was not affecting the body weight in mice, though glucose intolerance and insulin resistance were commenced [49]. Only after three month of high-fat feeding, did also the body weight respond to the increased nutritional pressure. This suggests, in order to thoroughly investigate the effects of resveratrol treatment on body weight, longer treatment periods are needed, which were unfortunately not possible in our study due to limitations of the pumping capacity of the osmotic mini-pump.

Finally, it was surprising to see that despite the GSIS was significantly increased by 29% compared to controls, the plasma glucose concentration was not lowered significantly 15 min after glucose administration (Fig 2A and 2C), which was also seen in the study by Nguyen *et al*. [10]. Thus, we speculate, despite lack of elevated fasting glucose and insulin levels, that LPS induces some subtle insulin resistance which only is revealed during a glucose challenge. A more sensitive method for assessing insulin sensitivity, like the euglycemic-hyperinsulinemic clamp, will probably be needed in order to study the degree of insulin resistance in more detail. Adiponectin expression has previously been described to be decreased by inflammation in murine adipocytes [50–52] and human subcutaneous adipose tissue [44, 53, 54]. We did see that the adiponectin expression was decreased (and partially rescued by resveratrol) in the subcutaneous but not the epididymal adipose tissue (Fig 4A). However, this effect of resveratrol was not translated into mature protein, where only a small non-significant decline by LPS of plasma adiponectin was seen (Fig 4A).

This paper adds the growing field concerning the role of LPS and endotoxemia in the development of metabolic diseases. We here demonstrate that low-dose LPS enhance GSIS without affecting the glucose concentration suggesting increased insulin resistance. Resveratrol dampened the effect on the LPS-induced hyperinsulinemia and specifically reduced inflammation in epididymal adipose tissue pointing towards a possible important involvement of this tissue for the effects on insulin resistance and insulin secretion as a result of metabolic endotoxemia. Given the beneficial effect of resveratrol on specifically visceral adipose tissue, makes it an interesting candidate in ameliorating inflammation as seen in obesity and metabolic syndrome.

## Supporting Information

S1 Fig Western blot analysis on skeletal muscle. AKT (isoform 2), AS160, glycogen synthase, cytochrome c, pyruvate dehydrogenase, SDHA and HSP60 were investigated by Western blot analysis. However, no significant alterations in protein expression were induced by resveratrol and/or LPS. Data are presented as means ± SEM.
(TIF)

S1 Table Primary and secondary antibodies used for Western blot analysis. (DOCX)
