# Supplementary figures and images for: LPS-Enhanced Glucose-Stimulated Insulin Secretion Is Normalized by Resveratrol
Source: PLoS One. 2016 Jan 11;11(1):e0146840. doi: 10.1371/journal.pone.0146840 (PMC4709071; doi:10.1371/journal.pone.0146840)

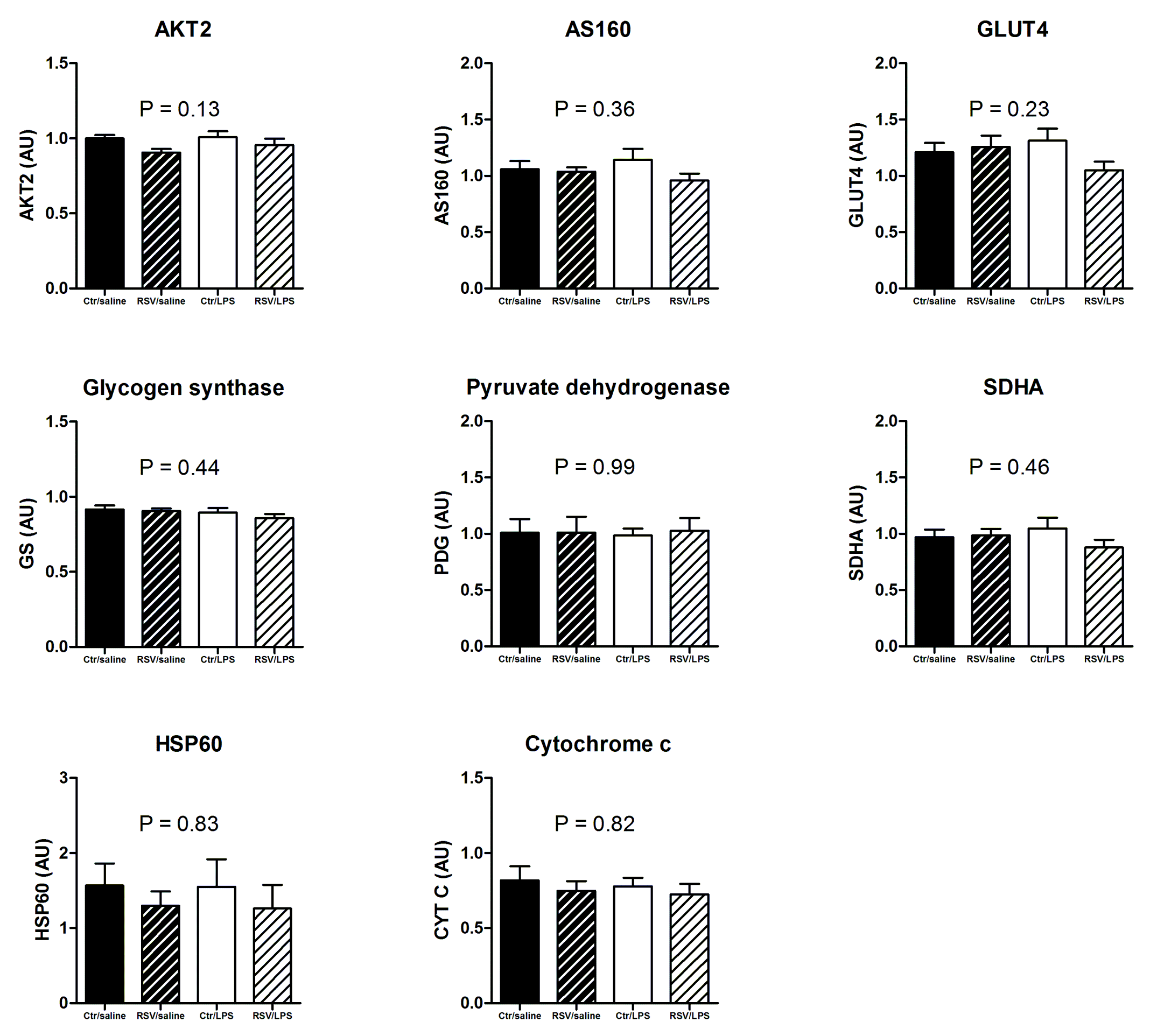

Supplement: S1 Fig — AKT (isoform 2), AS160, glycogen synthase, cytochrome c, pyruvate dehydrogenase, SDHA and HSP60 were investigated by Western blot analysis. However, no significant alterations in protein expression were induced by resveratrol and/or LPS. Data are presented as means ± SEM. (TIF) [file pone.0146840.s001.tif]
