# Supplementary material for: LPS-Enhanced Glucose-Stimulated Insulin Secretion Is Normalized by Resveratrol
Source: PLoS One. 2016 Jan 11;11(1):e0146840. doi: 10.1371/journal.pone.0146840 (PMC4709071; doi:10.1371/journal.pone.0146840)
Supplement: S1 Table — (DOCX) [file pone.0146840.s002.docx]

| Target | Host | Dilution | Source |
| --- | --- | --- | --- |
| Primary |  |  |  |
| AKT (isoform 2) | Rabbit | 1:1000 | Cell Signaling (3063) |
| AS160 | Rabbit | 1:3000 | Cell Signaling (07-741) |
| Cytochrome c | Rabbit | 1:1000 | Cell Signaling (4280) |
| GLUT4 | Rabbit | 1:1000 | Millipore (07-1404) |
| Glycogen synthase | Rabbit | 1:3000 | Cell Signaling (3886) |
| HSP60 | Rabbit | 1:1000 | Cell Signaling (12165) |
| PDH | Rabbit | 1:2000 | Cell Signaling (3205) |
| SDHA | Rabbit | 1:1000 | Cell signaling (11998) |
| Secondary |  |  |  |
| Anti-rabbit | Goat | 1:10000 | Santa Cruz (sc2054) |
